# Rosas’s Operation for Cataract

**Published:** 1843-10

**Authors:** 


*Rosas' Operation for Cataract.*—"Rosas is a dexterous and steady operator. In his extraction the patient is seated on a low stool, with the head placed obliquely to the light, and resting against the breast of an assistant who raises the upper lid, while the operator depresses the lower with the middle and forefinger in the usual manner. He makes the downward section with a knife somewhat different from that of Beer, as originally used by him, and figured in his work in 1830. This knife is much shorter in the blade than Beer's, its posterior edge (or back) is also sharp and slightly convex. Holding it between the thumb, and the index and middle fingers, the ring finger bent unto the hollow of the hand, and the little one resting on the cheek bone, he introduces the point at a right angle with the cornea (to prevent its catching in its layers), a little above the transverse axis of the eye, and having entered the anterior chamber, he alters the position of the instrument by depressing its handle towards the temporal fossa, and thus brings the surface of the blade on the same plane with that of the iris. Having passed it rapidly through the chamber and made the counter punctuation, so that a full quarter of an inch of the point has passed through the inner margin of the cornea, he then *draws* it slowly downwards, and slightly outwards, and so completes the section. If the case is one of double cataract he makes the corneal section, and concludes the operation in the second eye before he extracts the lens of the first. He opens the capsule with a Langenbeck's needle, sharpened on its concave edge, and extracts the lens by gently pressing on the upper portion of the cornea with the flat of the needle.

"The object aimed at in having the back of the knife curved is, to give it shortness as well as breadth, and thus avoid pricking the side of the nose; and its posterior sharp edge is to permit of cutting upwards as well as downwards, and thus not only pass through the cornea with greater facility, but also enable the operator to extend the incision upwards if the original punctuation is too low. Another reason assigned by the inventor of this knife is, that its blade by being sharp at both sides, and forming in its section a compressed ellipse, permits less escape of the aqueous fluid in passing through the chamber, than the ordinary instrument.

"In this manner Rosas operates with the most marked success; but in other hands, especially beginners, his method and instruments are open to many objections. The insertion of the knife at right angles with the cornea, is very liable to transfix the iris, and by twisting the cornea itself, renders its further insertion less smooth and easy, and its cutting back endangers both sclerotic and iris, especially in turning its lower edge outward when completing the incision; and when the iris happens to roll over the back of the knife, it cannot be pressed off with the same facility as when the posterior part is blunt; should the point of the knife get entangled in the iris, he withdraws it and re-introduces it in another place; if the corneal opening is too small he enlarges it with a Daviel's scissors.—*Wilde's Austria.—Bullet. Med. Sci.*

